# Modeling PrP^Sc^ Generation Through Deformed Templating

**DOI:** 10.3389/fbioe.2020.590501

**Published:** 2020-10-06

**Authors:** Giovanni Spagnolli, Marta Rigoli, Giovanni Novi Inverardi, Yaiza B. Codeseira, Emiliano Biasini, Jesús R. Requena

**Affiliations:** ^1^Department of Cellular, Computational and Integrative Biology, Centre for Integrative Biology, University of Trento, Trento, Italy; ^2^Dulbecco Telethon Institute, University of Trento, Trento, Italy; ^3^CIMUS Biomedical Research Institute, University of Santiago de Compostela-IDIS, Santiago de Compostela, Spain

**Keywords:** deformed templating, PrP^Sc^, PrP^Sc^ structure, PrP amyloid, 4RβS, PIRIBS, molecular dynamics

## Abstract

Deformed templating is the process by which self-replicating protein conformations with a given cross-β folding pattern can seed formation of an alternative self-replicating state with different cross-β folding pattern. In particular, uninfectious but propagative PrP amyloid can transform into a *bona fide* infectious conformer, PrP^Sc^ through deformed templating. The process can take many rounds of replication (if taking place *in vitro*) or even several passages of the evolving PrP conformers through successive brains if *in vivo*, through experimental transmission. In all cases, deformed templating involves a forced conversion in which there is a mismatch between the template and the substrate and/or the templating environment, typically a recombinant PrP amyloid, adept at converting recombinant PrP under denaturing conditions (e.g., presence of chaotropic agents), encountering a glycosylated, GPI-anchored PrP^C^ substrate under physiological conversion conditions. Deformed templating is characterized by emergence of intermediate conformers that exhibit biochemical characteristics that are intermediate between those of the initial PrP amyloid and the final PrP^Sc^ conformers. Here, we took advantage of the recent elucidation of the structure of a PrP amyloid by cryo-EM and the availability of a physically plausible atomistic model of PrP^Sc^ that we have recently proposed. Using modeling and Molecular Dynamics (MD) approaches, we built a complete molecular modelization of deformed templating, including an atomistic model of a glycosylated intermediate conformer and a modified model of PrP^Sc^. Among other unanticipated outcomes, our results show that fully glycosylated PrP can be stacked in-register, and how 4-rung β-solenoid (4RβS) PrP architectures can share key structural motifs with parallel-in register intermolecular sheet (PIRIBS) PrP amyloids. Our results shed light on the mechanisms of prion replication.

## Introduction

Generation of the first synthetic prions in 2004 was a feat that changed the field of prion research (Legname et al., 2004). Synthetic prions were a logical corollary of the prion hypothesis that had been proposed about 20 years earlier by Stanley Prusiner (Prusiner, 1982). If mammalian prions were but a conformational variant of the mammalian protein PrP^C^, it followed that it would (and should) be possible to generate such conformation *in vitro*. However, the exact conditions, and co-factors needed to produce such transformation in the test tube were not obvious, and thus numerous attempts to produce recombinant prions failed. Legname, Baskakov, and cols. reasoned that given that PrP^Sc^ often appeared in the form of amyloid fibrils (Merz et al., 1981; DeArmond et al., 1985), converting recombinant PrP (recPrP) into amyloid fibers *in vitro* would be the most likely strategy to succeed. It should be noted that at the time there was a controversy as to whether the amyloid nature of PrP^Sc^ fibers was an intrinsic feature of the infectious prionic agent or just an epiphenomenon (Prusiner, 1998). In other words, these researchers reasoned that even if the amyloid they prepared was not the infectious agent *per se*, PrP amyloid preps would likely contain the infectious conformation even if as a minor component (Legname et al., 2004). Their success in unequivocally transmitting prion disease to a group of mice using PrP amyloids generated from recombinant protein and a set of simple chemicals alone, and thus completely free of any nucleic acid, was the definitive experimental proof of the prion hypothesis (Legname et al., 2004). However, this result was not exempt of controversy: the animals used in the experiment were transgenic mice expressing a truncated version of PrP, MoPrP 89–230, in large quantities (∼16x the expression seen in wt animals). This led some critics to argue that perhaps the PrP amyloid prep had not actually produced a prion disease, but rather, accelerated spontaneous development of a genetic form of neurodegeneration induced by overexpression of an aberrant form of PrP.

These objections were definitively laid to rest a few years later when Ma and cols. generated a recPrP^Sc^ capable of infecting wild type mice with an attack rate of 100% and incubation times similar to those typical of brain-derived PrP^Sc^ (Wang et al., 2010). These authors used a modified version of protein misfolding cyclic amplification (PMCA), involving addition of co-factors to the conversion mixture, to generate their recPrP^Sc^, rather than simple amyloid fibrillization (Wang et al., 2010). The term recPrP^Sc^ aptly expresses the fact that their conformation is believed to be the same or virtually the same as that of brain PrP^Sc^, i.e., the mammalian prion, whereas to describe the infectious conformer(s) prepared by Legname, Baskakov, and cols. the more conservative and generic “infectious PrP amyloid” is preferable. More recently, numerous recPrP^Sc^ preps have been described (Piro et al., 2011; Deleault et al., 2012; Fernández-Borges et al., 2018; Eraña et al., 2019), generated by different PMCA-based methods and relying on different sets of co-factors. Rec-PrP^Sc^ has in fact become a very promising material for structural studies, as it opens infinite possibilities of labeling, including with stable isotopes for solid state NMR (ssNMR) studies (Sevillano et al., 2018; Martín-Pastor et al., 2020). Preliminary studies suggest that all these infectious recPrP^Sc^ variants are structurally similar *inter se* but different from the simpler PrP amyloid preps obtained by shaking recPrP under denaturing conditions (Tycko et al., 2010; Baskakov et al., 2019). One obvious difference is the extension of the β-sheet-rich core, which in PrP^Sc^ typically spans from ∼89 to 230 (mouse numbering), whereas in simpler PrP amyloids it spans only from ∼160/170 to 230 (Bocharova et al., 2005; Lu et al., 2007; Smirnovas et al., 2009, 2011; Tycko et al., 2010). This in turn results in larger Proteinase K (PK) resistant cores in infectious recPrP^Sc^ conformers as compared with classic PrP amyloid (Bocharova et al., 2005; Lu et al., 2007; Smirnovas et al., 2009, 2011).

Shortly after the accomplishment of prion disease transmission with recPrP amyloids, Baskakov and associates made an unexpected discovery: while wt Syrian hamsters (Sha) inoculated with recombinant ShaPrP amyloid did not develop any clinical signs of prion disease, some of them accumulated abnormally folded forms of PrP, and inoculation of their brain homogenates to wt animals in a second passage induced a full-fledged prion disease (Makarava et al., 2010). These authors have prepared over time different variants of PrP amyloid, all behaving in a similar fashion, with a silent propagative phase followed by emergence of clinical disease in second or even third passage (Makarava et al., 2011, 2012, 2015, 2016; Makarava and Baskakov, 2012; Klimova et al., 2015). These studies have revealed that under most experimental conditions, upon first passage, a propagative, misfolded PrP species emerges that exhibits a peculiar, atypical pattern of PK-resistance. Namely, its main PK-resistant core spans from a position around ∼160 to the C-terminus and excludes the epitope recognized by antibody 3F4 (109–112) but includes the epitope of antibody SAF-84 (160–170). Such PK-resistant core is also characteristic of the PrP amyloid used for inoculation, and its size is related to the span of the PrP amyloid β-sheet-rich core already mentioned. The atypical, propagative PK-resistant PrP conformer is mostly monoglycosylated, although small fractions of diglycosylated and non-glycosylated conformer are also seen. With successive passages, classic PrP^Sc^ starts to emerge, eventually outcompeting the atypical propagative PK-resistant PrP conformer (Makarava et al., 2011, 2012, 2015, 2016; Makarava and Baskakov, 2012; Klimova et al., 2015). Careful experiments were carried out to discriminate between two possibilities: (a) minute amounts of PrP^Sc^ are present from the beginning in the first passage or (b) the atypical, propagative PK-resistant PrP conformer precedes PrP^Sc^, which originates by a slow and progressive conversion of the former into the latter conformer. Baskakov and colleagues ruled out experimentally the first possibility, for example, with extremely sensitive PMCA-based analyses that would have detected even a small handful of PrP^Sc^ molecules (Makarava and Baskakov, 2012; Makarava et al., 2012). Therefore, they coined the term “deformed templating” to refer to the second process, which they demonstrated to be taking place (Makarava and Baskakov, 2012; Makarava et al., 2012). Furthermore, these authors proposed a wider application of the term, which was defined as defined as a process by which self-replicating protein conformational states with a given cross-β folding pattern can seed formation of an alternative self-replicating state with different cross-β folding pattern. Of note, deformed templating also explains seeding of amyloid fibrils (presumably non-infectious) by PrP^Sc^ in real time quacking-induced conversion (RT-QulC) assays, which could be viewed as the opposite process.

Very recently, Wang et al. (2020) used cryo-electron microscopy and reconstruction techniques to decipher the structure of classic recombinant PrP fibers and used the experimental data to build an atomic structural model of this PrP conformer. On the other hand, we have built in the past an atomic model of PrP^Sc^ which is also based on cryo-EM data, albeit of much lower resolution (Vázquez-Fernández et al., 2016) and incorporates all other available experimental structural constraints (Spagnolli et al., 2019). Our model is the only one compatible with the most recent low-resolution experimental constraints and it is stable when challenged with plain MD simulations (Spagnolli et al., 2019). We reasoned that these two models offer a starting and a final point that could be used to build and test a molecular model of deformed templating.

We therefore employed a set of bioinformatic tools and approaches to develop a molecular mechanism of deformed templating. We present here our results.

## Materials and Methods

### Modeling the Glycosylated PK-res PrP Candidate

The atomic coordinates for the human PrP amyloid characterized by Wang et al. (2020) were retrieved from PDB 6LNI (containing five PrP monomers arranged as a parallel-in-register-β-sheet). Protein topology was generated in Gromacs 2018 (Van Der Spoel et al., 2005) using Amber99SB-ILDN forcefield (Lindorff-Larsen et al., 2010). Asparagines N_181_ and N_197_ of each PrP monomer were glycosylated. The glycosylation procedure was carried out using doGlycans (Danne et al., 2017), a python-based tool that generates carbohydrate structure of glycoproteins, provided the carbohydrates sequence and the protein structure as input. The glycans employed are composed by four *N*-acetylglucosamines, 3 mannoses, 2 galactoses, 1 fucose, and 2 sialic acids (GlcNAc_4_Man_3_Gal_2_FucNeuNAc_2_) (Baskakov, 2017). The structure of the glycosylated fibril was visually inspected and torsional angles of the carbohydrates were adjusted to avoid extensive overlap between the sugar chains. The resulting structure was solvated in water (TIP3P) and ions (NaCl) at physiological concentration (150 mM). The solvated molecule was minimized using the steepest descent algorithm, in four steps. The first two steps were performed with position restraints on all protein atoms with force tolerance equal to 1000 kJ/(mol⋅nm) and then 500 kJ/(mol⋅nm), while the 3rd and the 4th steps were executed without restraints, with the same aforementioned tolerance values. The absence of steric clashes was evaluated using the Clashes/Contact Tool in UCSF Chimera. To generate the glycosylated decamer, two fully glycosylated pentamers were stacked. The system was then energy minimized following the aforementioned protocol.

### Building the Deformed-Templating-Compatible-4-Rung β-Solenoid (4RβS) Mouse PrP^Sc^ Model

The stretch consisting of residues 181–208 (human numbering), retrieved from PDB 6LNI, was used as a template to build each rung of the 4RβS model. The threading scheme was obtained by considering different experimental constraints: PK cleavage sites, indicating residues likely excluded from the resistant core of the protein, mass spectrometry studies, revealing the presence of a disulfide bond between the two cysteines of PrP sequence, and the solvent exposure of the glycosylated side-chains that must be capable of accommodating the bulky glycans (Spagnolli et al., 2019). Residue numbering was switched to the mouse sequence and, in the different rungs, the amino acids were swapped to the target ones, to match the proper threading, using UCSF Chimera (Pettersen et al., 2004). Side-chains rotamers were retrieved from the Dunbrack’s library. Protein loops were built using MODELLER (Sali and Blundell, 1993). Protein topology was generated in Gromacs using Amber99SB-ILDN forcefield and the resulting structure was solvated in water (TIP3P) and ions (NaCl) at physiological concentration (150 mM). The system was then energy minimized using the steepest descent algorithm with position restraints (force constant of 10000 kJ⋅mol^–1^⋅nm^–2^) on no-hydrogen atoms of backbone residues involved β-strands formation. A subsequent step of energy minimization was performed by removing the position restraints from all-atoms (a force tolerance of 200 kJ⋅mol^–1^⋅nm^–1^ was set for energy minimizations). At the end of this step, Ramachandran outliers and low-probability rotamers were corrected using Coot (Emsley et al., 2010). The absence of steric clashes was evaluated using the Clashes/Contact Tool in UCSF Chimera. 4RβS tetramer was built by stacking four monomers in a head-to-tail fashion followed by the previously reported energy minimization and refinement protocol. The glycosylated deformed-templating-compatible-4RβS tetramer was build using the same procedure employed to construct the glycosylated recPrP amyloid.

### Molecular Dynamics Simulations and Analysis

The following protocol was employed for the glycosylated PrP amyloid, the non-glycosylated PrP amyloid and the 4RβS PrP^Sc^ model: the energy minimized system was subjected to 1 ns of NVT equilibration at 300 K by employing the V-rescale thermostat (Bussi et al., 2007). Then, NPT equilibration was performed by employing the V-rescale thermostat and the Perrinello-Rahman barostat (Parrinello and Rahman, 1981) at 300 K and 1 Bar for 1 additional ns. These two steps were carried out using positional restraints, with force constant of 1000 kJ/(mol⋅nm^2^), on no-hydrogen atoms. Subsequently, 20 ns of molecular dynamics simulations (300 K, 1 Bar) were performed by introducing distance restraints between backbone atoms (amide H and O) involved in hydrogen bonding for β-strands formation (for more details, see Spagnolli et al., 2019). At the end, distance restraints were removed and the trajectory was extended to additional 100 ns of plain MD. This procedure was repeated three times for the three starting structure, yielding three independents 100 ns trajectories each. The simulations were performed using Gromacs 2018. Trajectories were then analyzed with VMD 1.9.2 (Humphrey et al., 1996), in particular, RMSD was computed using the “RMSD Trajectory Tool,” while the β-strand content was evaluated with the “Timeline Tool.” Graphs and images were generated with Matplotlib (Hunter, 2007) and UCSF Chimera, respectively.

## Results

### Building of an Atypical Propagative PK-Resistant PrP Candidate

**FIGURE 1:**
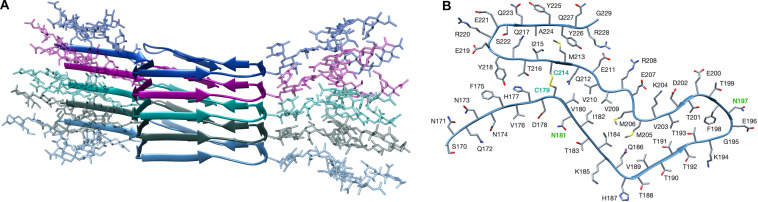
Structural model of a fully glycosylated human PrP amyloid. **(A)** The structure of the PrP amyloid is represented as a cartoon, each of the monomer is depicted in different colors. Glycans are shown with sticks representation and colors matching the one of the linked PrP monomer. No steric clashes are present in the structure. **(B)** Top view of a single PrP amyloid monomer, cysteine labels are depicted in cyan, showing the disulfide bond between C_179_ and C_214_. Labels of glycosylated asparagines (N_181_ and N_197_) are shown in green. The structure of the non-glycosylated human PrP amyloid was retrieved from PDB 6LNI.

As noted by Baskakov and associates, the PK fragmentation pattern of the atypical, propagative PK-resistant PrP conformer that appears at the beginning of the deformed templating process is essentially identical to that of the original PrP amyloid inoculated (Makarava et al., 2012). This suggests that their architectures are likely to be very similar. However, one major difference between the two is that the atypical, propagative PK-resistant PrP conformer is glycosylated (mostly mono-glycosylated) and displays a GPI-anchor. Therefore, we first aimed at building a glycosylated version of the recombinant PrP amyloid whose architecture has recently been elucidated by Wang et al. (2020). For this, we generated a GlcNAc_4_Man_3_Gal2FucNeuNAc_2_ glycan (Supplementary Figure S1), which is representative both in size, complexity and branching, of the most abundant glycans found in PrP^C^ and PrP^Sc^ (Rudd et al., 1999, 2001; Pettersen et al., 2004) and attached two of them *in silice* to the PrP amyloid model of Wang and colleagues (PDB 6LNI) at asparagines N_181_ and N_197_. Based on prior considerations (Terruzzi et al., 2020) we expected that it would be very difficult to stack more than two monomers of such putative conformer because of steric clashes; however, to our surprise, we were able to easily stack a pentamer of the diglycosylated structure, without any steric clashes (Figure 1).

### Molecular Dynamics Simulation of the Stability of the PIRIBS Atypical Propagative PK-Resistant PrP Candidate

**FIGURE 2:**
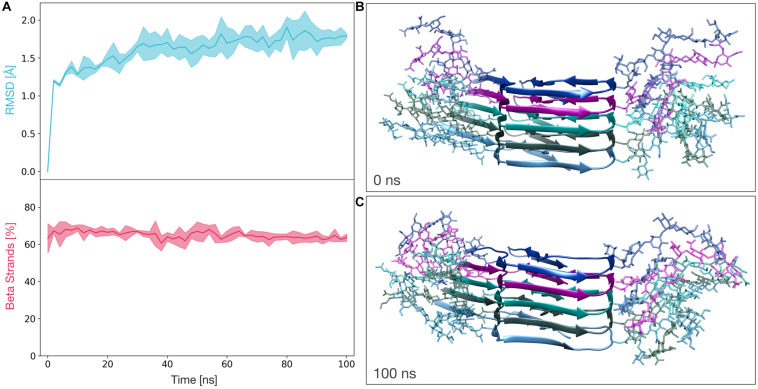
Molecular Dynamics simulations of the fully glycosylated PrP amyloid. **(A)** The graphs show the RMSD (above) and percentage of β-strands (below) as a function of the simulation time. The line and the filled curve indicate the mean and the standard deviation, respectively, computed on the three performed simulations of 100 ns each. **(B)** Representative snapshot extracted at the beginning of the MD simulations (*t* = 0 ns). **(C)** Representative snapshot extracted at the end of the MD simulations (*t* = 100 ns).

We next performed MD simulations to evaluate the stability of the glycosylated PK-resistant PrP candidate. This PIRIBS structure (constituted by five diglycosylated subunits) was subjected to three independents 100 ns molecular dynamics simulations. The stability of the model was then measured in terms of root mean squared deviation of atomic position from the initial state (RMSD) and β-strand content. For the entire length of the simulations, the average RMSD of the protein lies below 2 Å and the average percentage of β-strands remains greater than 60% (Figure 2A). These results indicate a significant stability of the model, and are comparable to those obtained for the non-glycosylated PrP amyloid (Supplementary Figure S2A).

### Building of a Modified 4RβS Atomic Model of PrP^Sc^, Compatible With Deformed Templating

**FIGURE 3:**
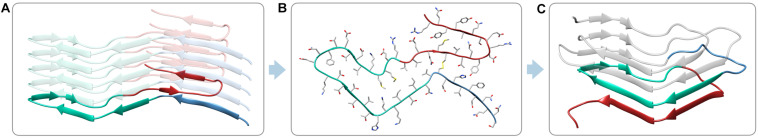
Building a deformed-templated-compatible 4RβS PrP^Sc^. Different stretches of PrP residues are depicted in different colors, based on the conformation displayed in the target 4RβS structure. The triangular core (N_181_-R_208_, human numbering) is depicted in aquamarine, the sequence at its N-terminus (S_170_-V_180_) is depicted in blue and the structure at its C-terminus (V_209_ to end) is depicted in red. **(A)** Representation of the colored sequences in a lateral view of recPrP. **(B)** Top view of the PrP monomer in the PIRIBS conformation with side-chains represented as sticks. **(C)** 4RβS model compatible with deformed templating.

**FIGURE 4:**
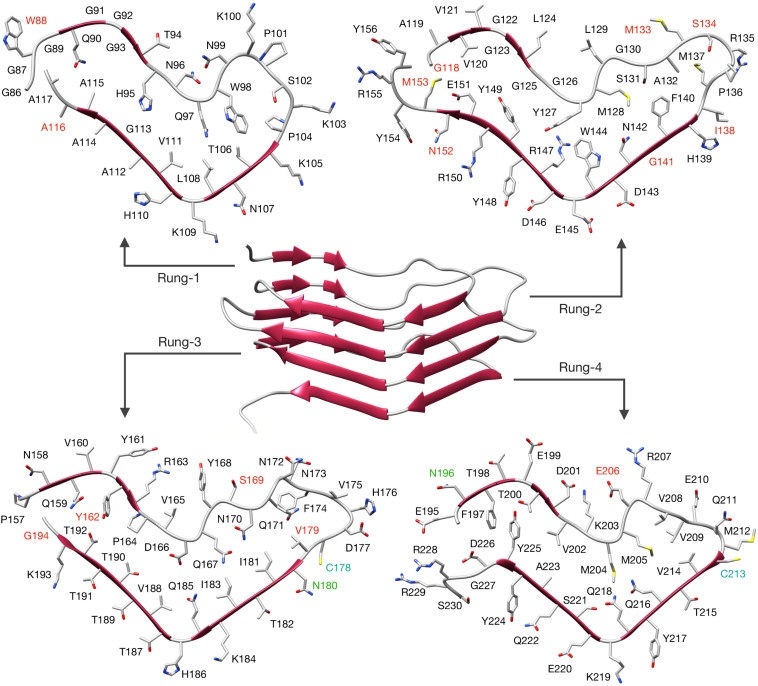
4RβS model of mouse PrP^Sc^ compatible with deformed templating. The structure of the deformed-templating-compatible PrP^Sc^ modeled as a 4RβS is shown in the figure center. Residues are depicted as sticks and displayed in each individual rung with labels in different colors. PK cleavage sites identified by mass spectrometry are labeled in red. Glycosylation sites are labeled in green and cystine in cyan. Residue numbering is relative to the mouse sequence. PDB coordinates of this model are available as Supplementary File S1.

Having solved the structure of a possible atypical propagative PK-resistant PrP conformer, we set out to model the core of the deformed templating process, i.e., transition from this early intermediate to a *bona fide* PrP^Sc^ conformer. We noticed that the cross-section of the PrP amyloid described by Wang et al. (2020) features an approximately triangular core spanning approximately N_181_ to R_208_ (Figures 3A,B). Such triangular core surface is relatively compatible with the triangular cross-section of our 4RβS PrP^Sc^ structural model (Spagnolli et al., 2019), therefore we built a modified version of PrP^Sc^ by using the architecture of this region to template the four rungs. The residues 170–180 (human numbering) at the N-terminal part of the triangular core are rearranged to form a loop, while the residues at the C-terminal side (209–231) are remodeled into a loop and two β-strands in the target 4RβS structure (Figure 3C). The rest of the 4RβS was built by considering the same experimental constraints employed to construct our previously proposed PrP^Sc^ model (Spagnolli et al., 2019). Due to poor availability of experimental constraints of human PrP^Sc^, we built the deformed-templating-compatible PrP^Sc^ model based on the mouse sequence. Importantly, the triangular core defined by residues 181–208, in the human sequence, and 180–207, in mouse one, share 100% identity. The resulting model (Figure 4), resembles our previously proposed one in many features: residues forming the inner core (T_106_-A_114_, Y_127_-L_129_, N_142_-Y_149_, and T_215_-Y_224_) are structurally conserved in the two putative PrP^Sc^ conformation (namely, residues pointing inward, or outward the hydrophobic regions are the same in both models); furthermore, important interactions are also shared between the two conformations, such as the buried salt-bridge between R_147_ and D_166_. Despite these common characteristics, the two models also display notable differences, such as the overall shape of the β-solenoid, the orientation of particular residues composing the resistant core (T_94_-N_99_, Q_167_-Y_168_, and Q_185_-H_186_) and the stretch composed of residues 198–201 that is excluded by the central part of the triangular core, with residues 203–206, occupying that region (methionines pointing inward). The two models do not exclude each other, and in fact the differences in solenoid shapes and threadings might constitute a molecular basis for strain differences. However, discussion of this possibility lies beyond the scope of this study.

Then, we built a tetrameric stack of diglycosylated 4RβS units (Supplementary Figure S3), showing a looser packing of glycans in this architecture, compared to the PIRIBS conformation.

### Molecular Dynamics Simulation of the Modified, Deformed Templating-Compatible, 4RβS Atomic Model of PrP^Sc^

**FIGURE 5:**
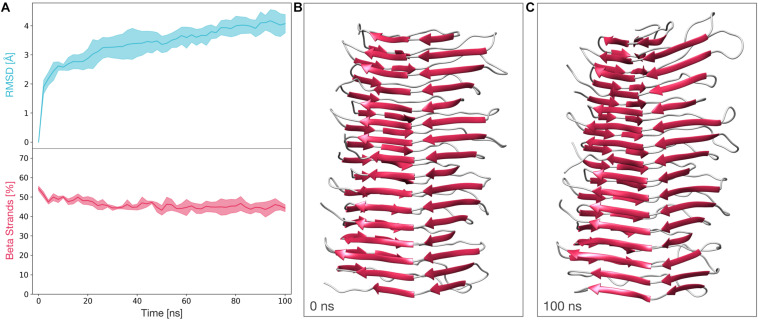
Molecular dynamics simulations of the deformed templating-compatible 4RβS model of PrP^Sc^. **(A)** The graphs show the RMSD (above) and percentage of β-strands (below) as a function of the simulation time. The line and the filled curve indicate the mean and the standard deviation, respectively, computed on the three performed simulations of 100 ns each. These results are compatible with the stability observed of the 4RβS model introduced in our previous work (Vázquez-Fernández et al., 2016). **(B)** Representative snapshot extracted at the beginning of the MD simulations (*t* = 0 ns). **(C)** Representative snapshot extracted at the end of the MD simulations (*t* = 100 ns).

We next subjected a tetrameric model of the deformed templating-compatible 4RβS to the same MD protocol we employed in Spagnolli et al. (2019). The stability of this structure was also assessed by computing the RMSD and the β-strands content along the trajectories. The average RMSD at the end of the simulation resulted to be ∼4 Å, while the average percentage of β-strands remains above 40% for the entire length of the simulations. These results show that the stability of this structure is comparable to the one observed in our previously proposed tetrameric model (Figure 5 and ref 28).

### Modeling Transition From the PIRIBS Atypical Propagative PK-Resistant PrP Intermediate to *Bona Fide* PrP^Sc^

**FIGURE 6:**
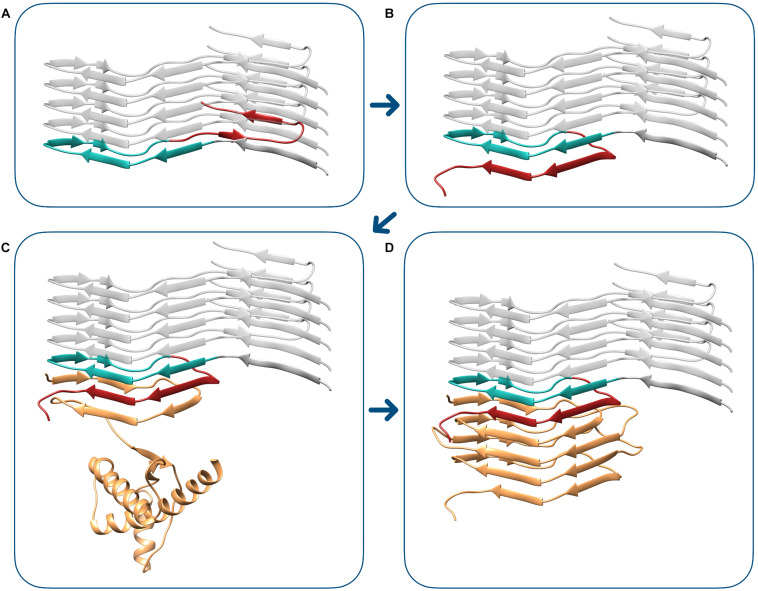
Modeling transition from the PIRIBS atypical propagative PK-resistant PrP intermediate to 4RβS PrP^Sc^. The deformed templating event begins with the conformational transition of the terminal PIRIBS monomer **(A)**, in which the C-terminal moiety (residues 208–230, mouse numbering), depicted in red, rearranges to engage intramolecular contacts with the central triangular core (residues 180–207), represented in aquamarine. This event generates an active-end, compatible with the propagation of the 4RβS architecture **(B)**. This terminal surface is now capable of templating the conversion of the unstructured region of PrP^*C*^ (depicted in orange), leading to the formation of the first rung of the β-solenoid, composed by residues 89–115 **(C)**. The completion of the conversion into 4RβS PrP^Sc^ **(D)** finally occurs by the sequential templating events of the subsequent rungs, as we have previously described (Vázquez-Fernández et al., 2016).

Finally, we elaborated a model of the transition between the PIRIBS atypical propagative PK-resistant PrP intermediate to *bona fide* PrP^Sc^. In the proposed mechanism, the triangular core of the PIRIBS architecture, defined by residues 180–207 (mouse numbering), plays the fundamental role of templating the formation of the β-solenoid rungs. The deformed templating begins with the structural rearrangement of the C-terminus of the fibril-end PrP monomer. In this conformational change, residues 182–192 of the triangular core engage hydrogen bonds with residues 215–225, resulting in the formation of two new β-strands. This structure now displays a C-terminal surface that is indistinguishable from the C-terminal rung (rung 4) of 4RβS, as residues 196–230 are now forming a complete rung 4. This active end is now capable of starting the templating of a 4RβS PrP^Sc^, that is achieved by a mechanism in which each rung templates the formation of the following one (as described in 28). A representation of the transition from the PIRIBS PrP intermediate (Figure 6A) to 4RβS PrP^Sc^ (Figure 6D) is shown in Figure 6.

## Discussion

Deformed templating, the process by which a classic PrP amyloid propagating in a brain or by PMCA eventually generates *bona fide* PrP^Sc^, is in our opinion of central importance in prion biochemistry. Templated propagation is the core of prion biology, and while substantial progress has been made to understand and model it at the molecular level (Spagnolli et al., 2019; Terruzzi et al., 2020), a complete understanding of it will not be achieved until a full experimental model of PrP^Sc^ is available. And while such an understanding is not achieved, critical issues of practical importance such as transmission barriers and the identification of PrP^Sc^-aimed therapies will be impossible. In this context, deformed templating provides an important constraint for the structure of PrP^Sc^ and the molecular process of its propagation: the structure and the process must be such that they are compatible with deformed templating. In other words, the structure of PrP^Sc^ must be such that with reasonable strains (deforming) it can be reached from a PrP amyloid as a starting point. And the fact that the structure of a PrP amyloid has been deciphered with a quasi-atomic resolution using cryo-EM offers a unique opportunity to challenge the PrP^Sc^ structural model, and the model of PrP^Sc^ propagation that we have proposed recently (Spagnolli et al., 2019).

Here, we provide a modelization of deformed templating that allows such challenging exercise and at the same time offers a plausible description of the process at the molecular level. As described in numerous studies from the Baskakov lab, the first step in deformed templating involves an intermediate PrP conformer with a pattern of PK-resistance that is strikingly similar to that of the original propagative PrP amyloid prepared *in vitro* and used to inoculate the experimental animals (Makarava and Baskakov, 2012; Makarava et al., 2012). In particular, the main PK-resistant fragment is C-terminal, spanning from ∼160 to 231. We reasoned that the most parsimonious explanation for this would be if the intermediate shares the same architecture as the initial amyloid: a PIRIBS one. However, it has been assumed in the past that such architecture does not allow stacking of the glycans (Baskakov and Katorcha, 2016; Baskakov et al., 2019). Still, we decided to explore this possibility, and to our surprise, a diglycosylated PrP amyloid core pentamer modeled from the structure recently solved by Wang et al. (2020) was stackable into a very stable PIRIBS (Figures 1, 2). Furthermore, this pentamer could be extended to a decamer, showing the physical plausibility of longer glycoslylated assemblies (Supplementary Figure S4). Therefore, when a theoretically non-infectious PrP amyloid is inoculated into a brain (Makarava et al., 2011, 2012, 2015, 2016; Makarava and Baskakov, 2012; Klimova et al., 2015), some of the glycosylated PrP^C^ molecules (in particular the mono-glycosylated ones) of the host can be templated and incorporated into the amyloid seeds (Figure 1). Why are monoglycosylated PrP^C^ units incorporated preferentially over di- and non-glycosylated ones (Makarava et al., 2011, 2012, 2015, 2016; Makarava and Baskakov, 2012; Klimova et al., 2015)? One key characteristic of this first templating event is that the PrP^C^ substrate units possess a GPI-anchor, and in fact conversion is likely to take place while PrP^C^ is attached to a cell membrane, as has been documented for PrP^C^ to PrP^Sc^ conversion in general (Godsave et al., 2008). This likely imposes constraints to the templating process, which must necessarily involve a partial/total unfolding of the C-terminal helices of PrP^C^. It is conceivable that while the two glycans can be well-accommodated in the final product, their joint presence might sterically hamper the process of approximation, attachment and templated refolding of a partially unfolded, membrane-attached PrP^C^ subunit. Thus, incorporation of non-glycosylated and monoglycosylated PrP^C^ units might be favored over incorporation of diglycosylated PrP^C^ units (Makarava et al., 2011, 2012, 2015, 2016; Makarava and Baskakov, 2012; Klimova et al., 2015). The low proportion of non-glycosylated subunits in the final oligomer would merely reflect their scarcity. Another fact that needs to be considered is that a portion of glycans (Rudd et al., 1999, 2001) are tri- and tetra-antennary, this is, bulkier than the model di-antennary glycan that we have used for the modeling. Incorporation of a diglycosylated PrP subunit with two of these bulkier than average glycans might therefore be less favorable. Finally, the presence of charged sialic acid in some glycan units adds ionic constrains that might also be an important factor in selective recruitment of monoglycosylated PrP^C^.

It should be noted that this first templating step, which involves the C-terminal region of PrP^C^, is very different from the second one, involving transition from the glycosylated propagative PK-resistant PrP intermediate to *bona fide* PrP^Sc^: in that case, conversion very likely begins at the flexible/intrinsically unfolded ∼90–120 segment of PrP^C^ (Spagnolli et al., 2019), already posed to be templated by any β-sheet-rich mold it encounters (from the perspective of the final main PK-resistant, β-sheet-rich core, the “N-terminus”). This segment of PrP^C^ is far apart from the glycans, which are much less likely to exert steric hindrances at the beginning of the conversion process, and therefore a selection of monoglycosylated over diglycosylated substrate PrP^C^ units.

According to our model, this second conversion step involves two sub-steps: first the C-terminus of the PrP unit at the amyloid templating edge (196–230) must rearrange (deform) to form a “PrP^Sc^ templating surface.” For this they have to engage in heterologous (i.e., non-PIRIBS) interactions and bonding for the first time. Once this sub-step is completed, the stage is set for the flexible “N-terminal” (∼90–120) stretch of a PrP^C^ substrate unit to be templated into *bona fide* PrP^Sc^ (Figure 6). Why is this intermediate rearrangement necessary, as opposed to a direct heterologous interaction of the “N-terminal” (∼90–120) stretch of an incoming PrP^C^ unit with the amyloid core of the atypical intermediate, or even the inoculum amyloid? We cannot provide an answer at this point, but the reason likely involves steric and energetic considerations.

We have based our modeling scheme on the human and murine sequences of PrP because our point of view is that deformed templating is a general process in PrP^Sc^ propagation. However, with minor changes it can be applied to ShaPrP, which was used in the majority of studies of the Baskakov lab. Furthermore, our modeling assumes that *bona fide* PrP^Sc^ is a 4RβS. Very substantial experimental evidence supports this view (Govaerts et al., 2004; Wille et al., 2009; Vázquez-Fernández et al., 2016; Wille and Requena, 2018; Spagnolli et al., 2019). However, an alternative PIRIBS model of PrP^Sc^ exists (Groveman et al., 2014). According to this model, PrP^Sc^ shares to some extent the C-terminal β-sheet-rich core of PrP amyloids, but the core extends with additional β-strands/loops up to position ∼90. While we currently do not support that model, we acknowledge that the matter is far from settled (Baskakov et al., 2019) and will not be until at least some ssNMR-based structural constraints become available. Therefore, we will briefly discuss our modeling-based findings in the context of that model. First of all, given that the propagative atypical intermediate we propose has a PIRIBS architecture, it is fully compatible with the model proposed by Groveman et al. (2014), although rearrangement of the tertiary structure and threading of some β-strands and connecting coils is necessary, as there are differences between the Groveman model and the structure of the PrP amyloid as deciphered by Wang et al. (2020). Once the glycosylated propagative atypical intermediate is formed, extension of the β-sheet-rich core in the same plane (perpendicular to the axis of growth of the stack) can take place easily. However, it has to proceed without any templating; rather, it would consist of a local rearrangement of the ∼90–170 flexible tails to collapse into each other to form additional β-sheets. The presence of cofactors such as lipids, polysaccharides, or polynucleotides acting as scaffolds/chemical chaperones, known to be critical for generation of *bona fide* PrP^Sc^ *in vitro* (Wang et al., 2010; Deleault et al., 2012; Eraña et al., 2019) would assist this non-templated, self-organizative process, until a definitive PrP^Sc^ templating surface is formed. Glycan stacking would not hamper the process, as shown by our modeling (Figures 1, 2). However, sufficient space must be allocated to both glycan stacks, and therefore any “glycan cleft” in the putative PrP^Sc^ PIRIBS model must be sufficiently wide to allow it. Nevertheless, it should be underscored that the 4RβS architecture makes incorporation of diglycosylated units simpler, since the distance between respective glycosylated residues is 19.2 Å instead of 4.8 Å, providing much more room.

## Conclusion

(1) We have modeled deformed templating, demonstrating how a simple PrP amyloid, with very low intrinsic infectivity, can evolve to adopt a *bona fide* PrP^Sc^ conformation. (2) We have found that a fully glycosylated PIRIBS amyloid stack is possible and stable; we propose that the atypical propagative intermediate that emerges initially during the deformed templating process is in fact a glycosylated PIRIBS amyloid. (3) We propose that such intermediate undergoes a conformational transition, whose nature we hypothesize at the atomistic level, to generate a PrP^Sc^ templating surface. (4) The constraints imposed by the necessity to allow for a deformed templating-based generation starting from a PIRIBS amyloid have led us to build a modified version of our previous 4RβS model of PrP^Sc^. (5) However, we acknowledge that deformed templating can be also modeled if PrP^Sc^ had a PIRIBS architecture; this is possible given that stacking of glycans in a PIRIBS is, as mentioned, possible. (6) Deformed templating blurs the categories of infectious vs. non-infectious PrP amyloids, possibly expanding and modulating the prion concept (Requena, 2020); in particular, we hypothesize that in the classic experiment by Legname, Baskakov and colleagues (Legname et al., 2004), deformed templating may have played a role in allowing a simple PrP amyloid to behave as a prion.

## Data Availability Statement

The raw data supporting the conclusions of this article will be made available by the authors, without undue reservation.

## Author Contributions

JR, EB, and GS designed the study. GS, MR, and GNI designed and carried out modeling and MD studies and analyzed the data. GS, MR, YC, EB, and JR integrated the data into a theoretical framework and elaborated conclusions. JR and GS wrote the manuscript. All authors revised and contributed to the final version of the manuscript.

## Conflict of Interest

The authors declare that the research was conducted in the absence of any commercial or financial relationships that could be construed as a potential conflict of interest.

## Supplementary Material

Supplementary Figure 1 Scheme of the model glycan. Both N181 and N197 of the glycosylated PrP amyloid are linked to this type of complex glycan. Different shapes in the figure represents different types of sugar moieties as described in the legend. The connectivity types between sugars is reported above (or below) the lines linking the shapes.

Supplementary Figure 2 Molecular Dynamics simulations of the non-glycosylated PrP amyloid. **(A)** The graphs show the RMSD (above) and percentage of β-strands (below) as a function of the simulation time. The line and the filled curve indicate the mean and the standard deviation, respectively, computed on the three performed simulations of 100 ns each. Structure for the simulations is retrieved from PDB 6LNI. **(B)** Representative snapshot extracted at the beginning of the MD simulations (*t* = 0 ns). **(C)** Representative snapshot extracted at the end of the MD simulations (*t* = 100 ns).

Supplementary Figure 3 Glycosylated structure of the deformed templating-compatible 4RβS model. The structure of the PrP^Sc^ 4RβS model is represented as a cartoon, each of the monomer is depicted in different colors. Glycans are shown with sticks representation and colors matching the one of the linked PrP monomer. In the 4RβS model the glycans are more spaced compared to the PIRIBS one (19.2 vs. 4.8 Å, as a distance between glycosylated residues). Thus, the 4RβS model can, in principle, accommodate fully glycosylated PrP^Sc^ monomers by introducing less strain compared to a PIRIBS PrP amyloid.

Supplementary Figure 4 Structure of a fully glycosylated decamer of PrP amyloid. The energy minimized decamer of recombinant PrP amyloid is represented as a cartoon, different colors are used to depict the monomers constituting the fibril. Carbohydrates are shown with sticks representation and colors matching the one of the attached PrP monomer.
