# Supplementary figures and images for: Modeling PrPSc Generation Through Deformed Templating
Source: Front Bioeng Biotechnol. 2020 Oct 6;8:590501. doi: 10.3389/fbioe.2020.590501 (PMC7573312; doi:10.3389/fbioe.2020.590501)

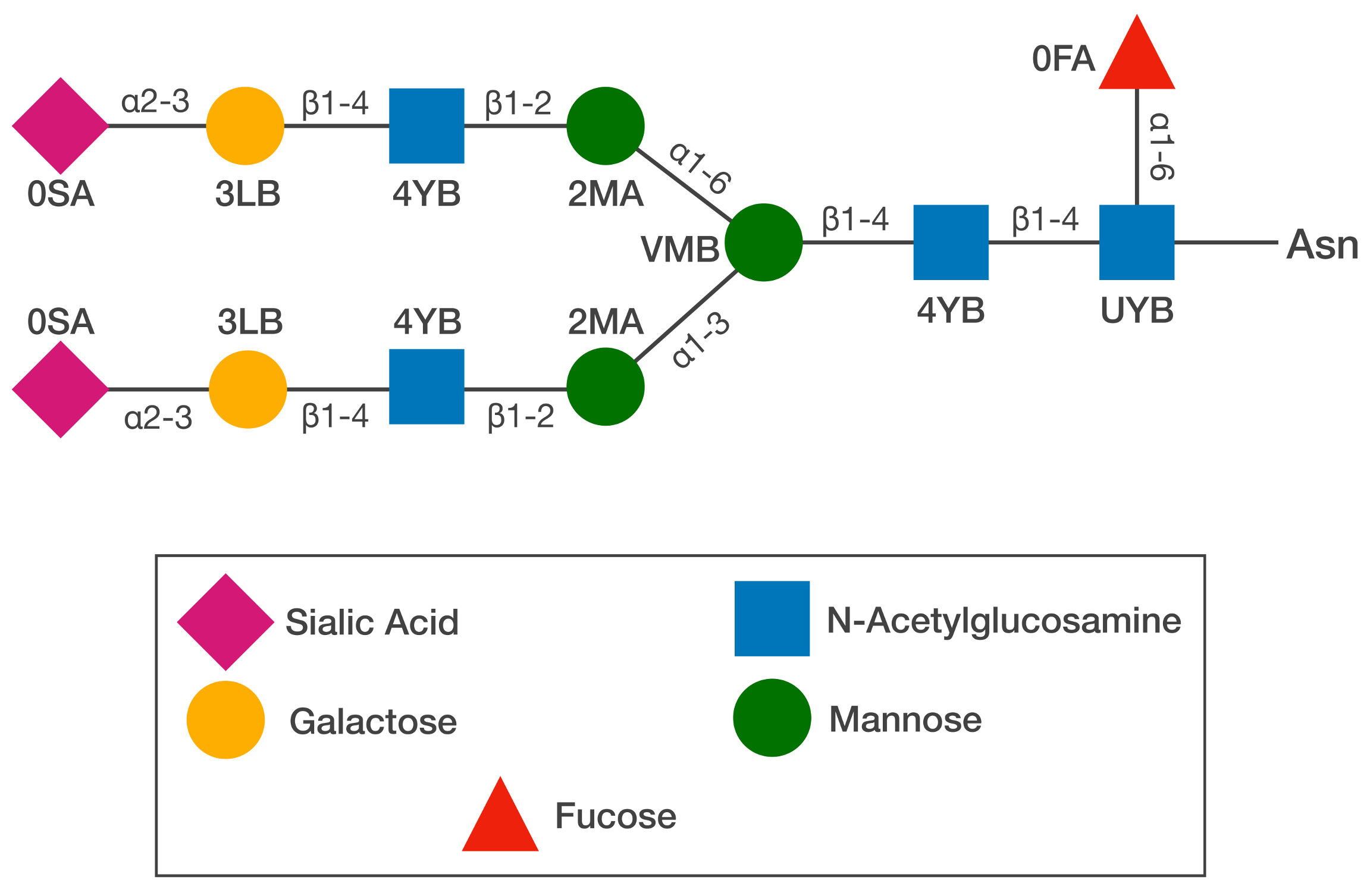

Supplement: Supplementary Figure 1 — Scheme of the model glycan. Both N181 and N197 of the glycosylated PrP amyloid are linked to this type of complex glycan. Different shapes in the figure represents different types of sugar moieties as described in the legend. The connectivity types between sugars is reported above (or below) the lines linking the shapes. [file Image_1.JPEG]

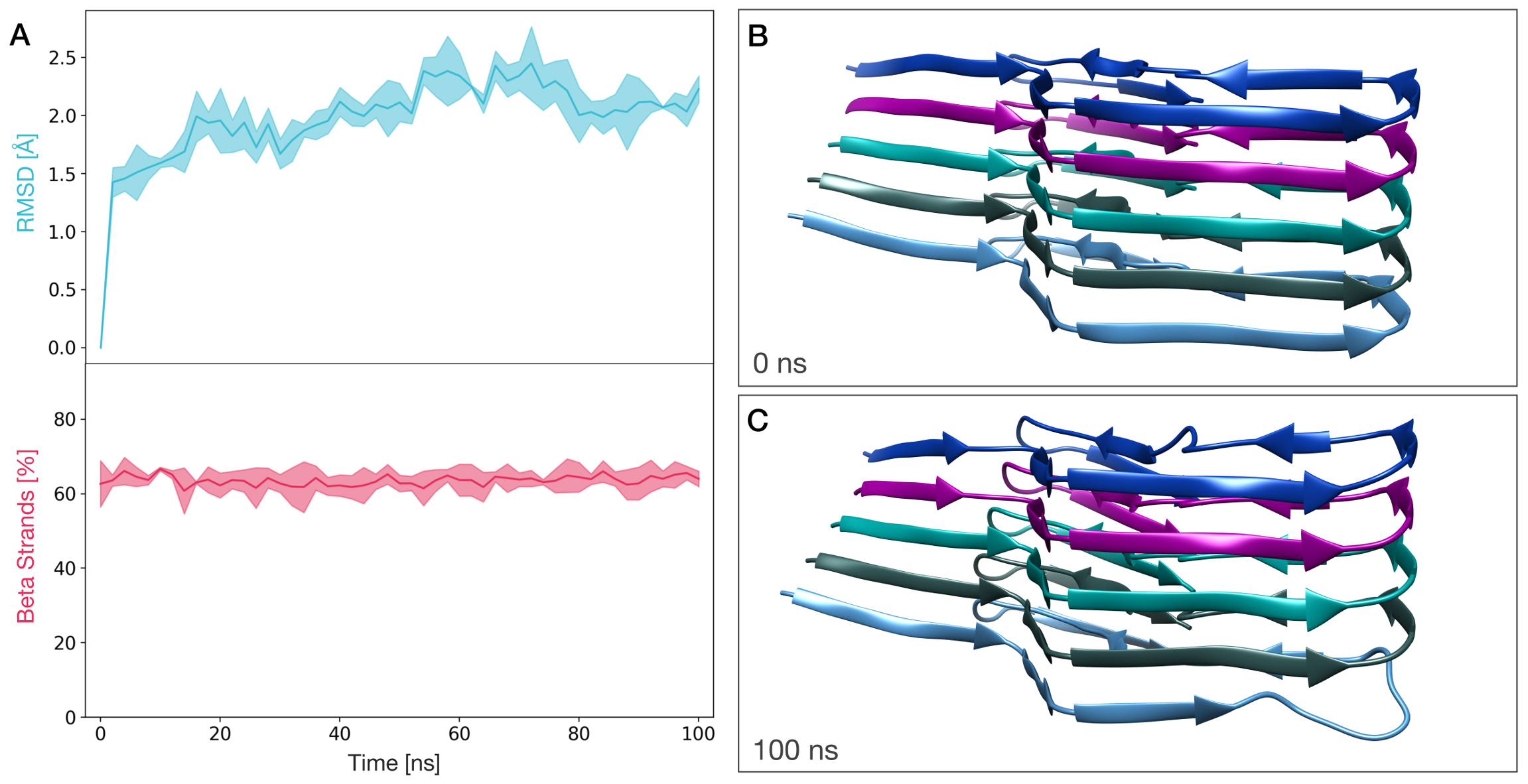

Supplement: Supplementary Figure 2 — Molecular Dynamics simulations of the non-glycosylated PrP amyloid. (A) The graphs show the RMSD (above) and percentage of β-strands (below) as a function of the simulation time. The line and the filled curve indicate the mean and the standard deviation, respectively, computed on the three performed simulations of 100 ns each. Structure for the simulations is retrieved from PDB 6LNI. (B) Representative snapshot extracted at the beginning of the MD simulations (t = 0 ns). (C) Representative snapshot extracted at the end of the MD simulations (t = 100 ns). [file Image_2.JPEG]

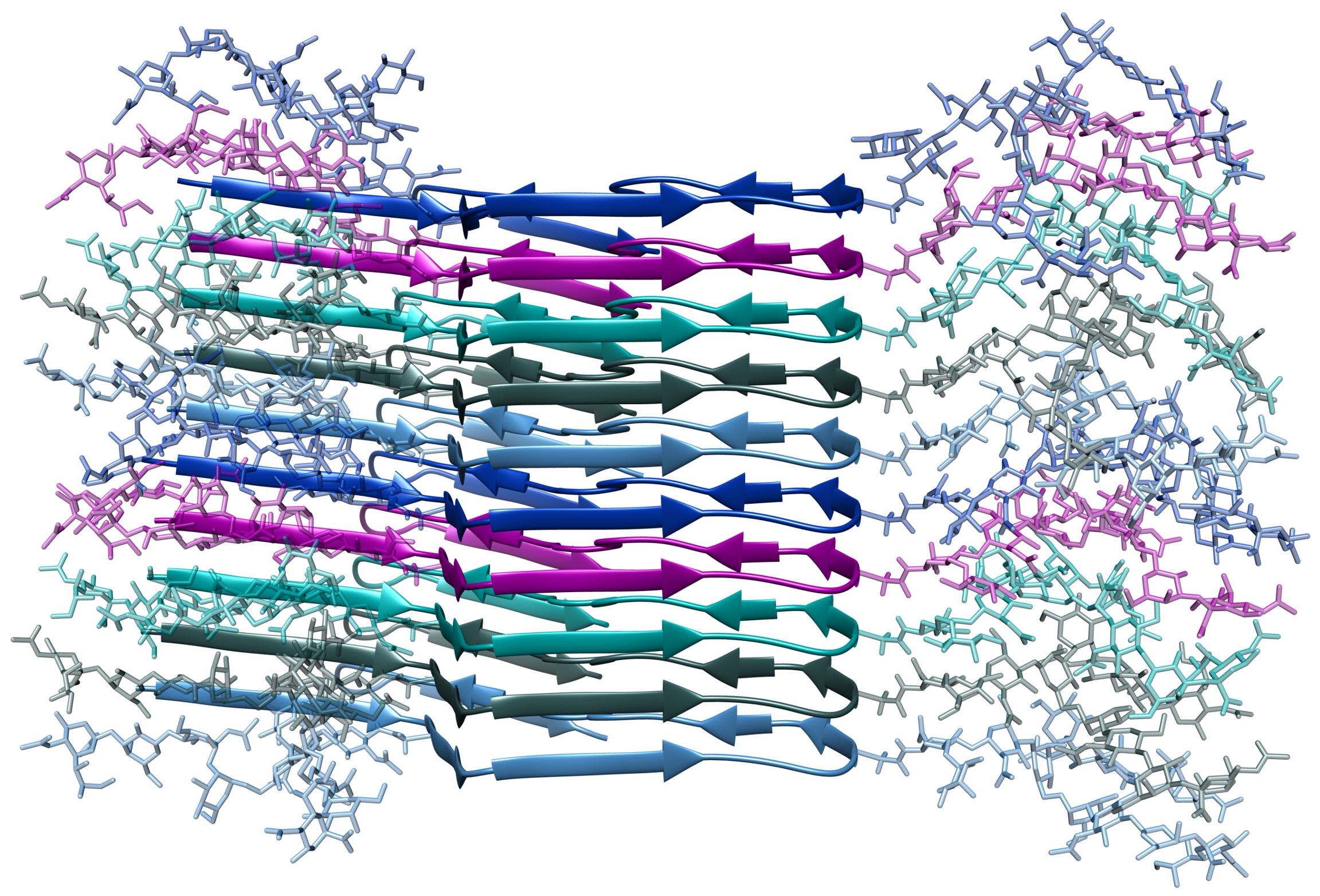

Supplement: Supplementary Figure 3 — Glycosylated structure of the deformed templating-compatible 4RβS model. The structure of the PrPSc 4RβS model is represented as a cartoon, each of the monomer is depicted in different colors. Glycans are shown with sticks representation and colors matching the one of the linked PrP monomer. In the 4RβS model the glycans are more spaced compared to the PIRIBS one (19.2 vs. 4.8 Å, as a distance between glycosylated residues). Thus, the 4RβS model can, in principle, accommodate fully glycosylated PrPSc monomers by introducing less strain compared to a PIRIBS PrP amyloid. [file Image_3.JPEG]

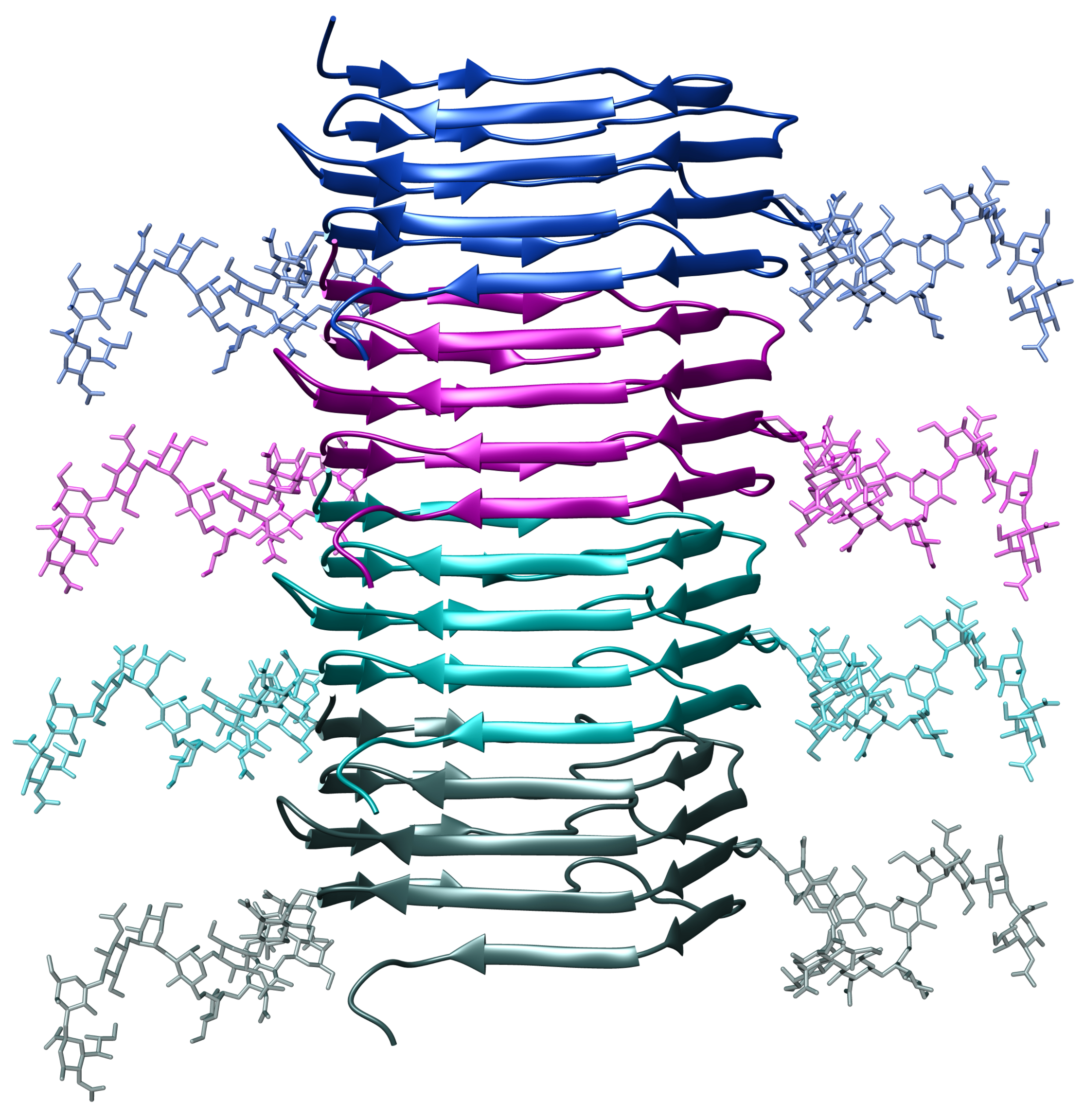

Supplement: Supplementary Figure 4 — Structure of a fully glycosylated decamer of PrP amyloid. The energy minimized decamer of recombinant PrP amyloid is represented as a cartoon, different colors are used to depict the monomers constituting the fibril. Carbohydrates are shown with sticks representation and colors matching the one of the attached PrP monomer. [file Image_4.JPEG]
